# Tumor-Associated Macrophage Polarization in Wilms’ Tumor After Neoadjuvant Chemotherapy

**DOI:** 10.3390/cancers18050810

**Published:** 2026-03-02

**Authors:** Karolina Malić Tudor, Sandra Zekić Tomaš, Ana Dunatov Huljev, Višnja Armanda Bogdan, Antonela Matana, Marin Ogorevc, Sven Seiwerth, Božo Krušlin, Jasminka Stepan Giljević, Ivana Kuzmić Prusac

**Affiliations:** 1 Department of Pediatrics, University Hospital of Split, 21000 Split, Croatia; kmalictudor@kbsplit.hr (K.M.T.); varmanda@kbsplit.hr (V.A.B.); 2 Department of Pathology, Forensic Medicine and Cytology, University Hospital of Split, 21000 Split, Croatia; adunatov@kbsplit.hr (A.D.H.); marin.ogorevc2@gmail.com (M.O.); ikuzprus@kbsplit.hr (I.K.P.); 3 School of Medicine, University of Split, 21000 Split, Croatia; 4 Faculty of Health Sciences, University of Split, 21000 Split, Croatia; antmatana@ozs.unist.hr; 5 Department of Pathology, School of Medicine, University of Zagreb, 10000 Zagreb, Croatia; sven.seiwerth@mef.hr (S.S.); bozo.kruslin@gmail.com (B.K.); 6 Ljudevit Jurak Clinical Department of Pathology and Cytology, Sestre Milosrdnice University Hospital Center, 10000 Zagreb, Croatia; 7 Department of Paediatric Oncology and Haematology, Children’s Hospital Zagreb, 10000 Zagreb, Croatia; jasminka.stepan@kdb.hr

**Keywords:** Wilms’ tumor, tumor-associated macrophages, macrophage polarization, M1 macrophages, M2 macrophages, tumor microenvironment, neoadjuvant chemotherapy, pediatric renal tumors, immunohistochemistry

## Abstract

**Background/Objectives**: Wilms’ tumor (WT) is the most common malignant renal tumor in childhood, and although survival rates are high, a subset of patients with high-risk disease remain prone to treatment resistance and relapse. Increasing evidence suggests that the tumor microenvironment, particularly tumor-associated macrophages (TAMs), may influence tumor behavior and therapeutic response. This study aimed to characterize M1 and M2 macrophage infiltration in WT treated with neoadjuvant chemotherapy according to the International Society of Pediatric Oncology (SIOP) protocol and to evaluate their association with clinicopathological features. **Methods**: Tumor tissue samples from 46 pediatric patients were analyzed using double immunohistochemical staining for CD80 (M1) and CD163 (M2) macrophages. TAM density and M1/M2 ratios were quantified in viable tumor areas and correlated with clinical, laboratory, and pathohistological parameters. **Results**: Total TAM counts were significantly higher in tumors with a volume ≥ 500 mL and in unifocal tumors. Both M1 and M2 macrophages were more abundant in larger tumors; however, M2 macrophages predominated overall. The M1/M2 ratio was positively associated with total TAM counts and was significantly higher in tumors with larger volume, elevated serum neuron-specific enolase, and increased creatinine levels. Regressive histological subtypes exhibited a higher M1/M2 ratio compared with other subtypes. No significant associations were observed between macrophage infiltration and tumor stage or risk group. **Conclusions**: WT treated with neoadjuvant chemotherapy demonstrates low overall macrophage infiltration with a predominance of M2 macrophages. TAM polarization appears to be associated with tumor burden and selected biochemical parameters, highlighting the potential relevance of macrophages as biomarkers and therapeutic targets in WT.

## Simple Summary

While survival rates for Wilms’ tumor are high, a subset of high-risk patients are prone to treatment resistance. Evidence suggests that tumor-associated macrophages may influence therapeutic response. The aim of our study was to characterize macrophage infiltration and polarization in Wilms’ tumors treated with neoadjuvant chemotherapy and to evaluate their association with clinicopathological features. Analysis of tumor samples from 46 patients revealed that larger tumors had a higher density of both M1 and M2 macrophages, with M2 macrophages predominating in general. The M1/M2 ratio was significantly higher in regressive histological subtypes, larger tumors, and in tumors of patients with elevated serum neuron-specific enolase and creatinine. No significant associations were observed between macrophage infiltration and tumor stage or risk group. Macrophage polarization in Wilms’ tumors appears to be associated with tumor burden and selected biochemical parameters, highlighting the potential relevance of macrophages as biomarkers and therapeutic targets.

## 1. Introduction

Wilms’ tumor (WT), also referred to as nephroblastoma, is the most common primary malignant renal tumor in children. Its annual incidence is relatively stable, with approximately 6–8 new cases per 1,000,000 children, and it accounts for nearly 90% of all renal tumors diagnosed in patients younger than 18 years [1,2,3,4,5]. Data from the Croatian Cancer Registry indicate that WT consistently represents 5–6% of all pediatric malignancies in Croatia [6,7].

The majority of WT cases occur sporadically; however, approximately 1% are inherited in an autosomal dominant manner [1,2]. WT is a genetically heterogeneous disease resulting from congenital or acquired biallelic mutations in genes essential for normal nephrogenesis, primarily tumor suppressor genes [8,9,10,11,12]. In addition, around 10% of WT cases are associated with congenital malformation syndromes [13,14,15].

Clinically, WT most commonly presents as a painless abdominal mass [16]. Gross examination typically reveals a solid or partially cystic, well-circumscribed nodular tumor with frequent areas of hemorrhage and necrosis, whereas calcifications are rare [17]. Histologically, WT may contain varying proportions of blastemal, stromal, and epithelial components; however, a classic triphasic pattern is not mandatory for diagnosis. According to the SIOP-RTSG classification, histopathological risk stratification is based on post-neoadjuvant chemotherapy morphology and is primarily determined by the presence or absence of diffuse anaplasia and the predominant histological subtype [18,19,20,21].

Based on histopathological features, WT is stratified into three risk categories: low risk (cystic partially differentiated nephroblastoma and completely necrotic tumors), intermediate risk (epithelial, stromal, mixed, or regressive subtypes and focal anaplasia), and high risk (blastemal subtype and diffuse anaplasia) [21,22].

Currently, two major therapeutic protocols are used worldwide. The International Society of Pediatric Oncology (SIOP) protocol, predominantly applied in Europe and several countries outside Europe, recommends preoperative (neoadjuvant) chemotherapy followed by surgical resection. Subsequent histopathological analysis enables accurate diagnosis and evaluation of treatment response by assessing the extent of tumor necrosis and the proportion of residual viable blastemal or anaplastic tissue [23]. In contrast, the Renal Tumors Committee of the Children’s Oncology Group (COG) protocol, used mainly in North America and formerly developed by the National Wilms Tumor Study Group (NWTSG), is based on primary surgical removal of the tumor, with adjuvant therapy guided by histological analysis of untreated tumor tissue [23,24].

With improving survival rates, increasing attention has been directed toward the long-term adverse effects of oncological treatment, highlighting the importance of treatment de-escalation strategies whenever oncologically feasible [25].

Solid tumors are complex biological systems composed of malignant cells and a dynamic tumor microenvironment (TME). The TME comprises stromal elements, neovascular structures, connective tissue, and immune cells, among which tumor-associated macrophages (TAMs) represent a dominant population [26,27,28,29]. Owing to their functional plasticity, TAMs are commonly categorized into classically activated, pro-inflammatory CD80-positive M1 macrophages and alternatively activated, immunosuppressive CD163-positive M2 macrophages [30,31,32].

The biological role of M1 and M2 macrophages in WT, as well as their impact on treatment response and clinical outcome, remains insufficiently defined. To date, only a limited number of studies have examined TAMs in WT treated according to the SIOP protocol, and these investigations were conducted in relatively small patient cohorts [33,34,35]. To the best of our knowledge, the present study is the first to systematically evaluate M1 and M2 macrophage populations in WT following preoperative chemotherapy in a representative cohort of pediatric patients.

The objective of this study was to identify and characterize CD80-positive M1 and CD163-positive M2 macrophages in post-chemotherapy WT specimens using double immunohistochemical staining, and to evaluate their association with established prognostic factors, including histological subtype, risk group, disease stage, patient age, and tumor volume, as potential indicators of immune response and neoadjuvant chemotherapy effectiveness. To our knowledge, this is the first study to comprehensively evaluate TAM polarization in WT specimens treated according to the SIOP-RTSG protocol, thereby addressing a clinically relevant gap in understanding the post-neoadjuvant TME.

## 2. Materials and Methods

### 2.1. Study Design

This retrospective cross-sectional study included pediatric patients diagnosed with WT and treated at the University Hospital of Split and the Children’s Hospital Zagreb between January 2000 and December 2017. All patients were managed according to the SIOP protocol. A total of 55 patients were initially identified. Nine patients were excluded from the analysis: four patients underwent primary nephrectomy due to severe tumor-related bleeding, and five patients were excluded because of incomplete clinical data. Consequently, the final study cohort consisted of 46 patients.

Clinical data, formalin-fixed, paraffin-embedded (FFPE) tumor samples, hematoxylin–eosin–stained slides, and corresponding histopathological reports were collected and analyzed. The follow-up endpoint was defined as 60 months after initiation of neoadjuvant chemotherapy or death, whichever occurred first.

Inclusion criteria comprised patients younger than 18 years with histologically confirmed WT treated with neoadjuvant chemotherapy followed by nephrectomy, availability of complete clinical and survival data, an accessible histopathological report, and adequately preserved FFPE tumor tissue. Exclusion criteria included non-Wilms pediatric renal tumors, lack of pathological tumor tissue evaluation, primarily surgically treated WTs, and incomplete clinical or survival data.

Ethical approval for the study was obtained from the Institutional Ethics Committee of the University Hospital of Split.

Macrophage infiltration was assessed by experienced perinatal pathologists who were blinded to all clinical data. Double immunohistochemical staining was performed on tumor tissue sections to identify macrophage subpopulations. CD80 and CD163 antibodies were used to detect M1 and M2 macrophage polarization, respectively.

**Figure 1:**
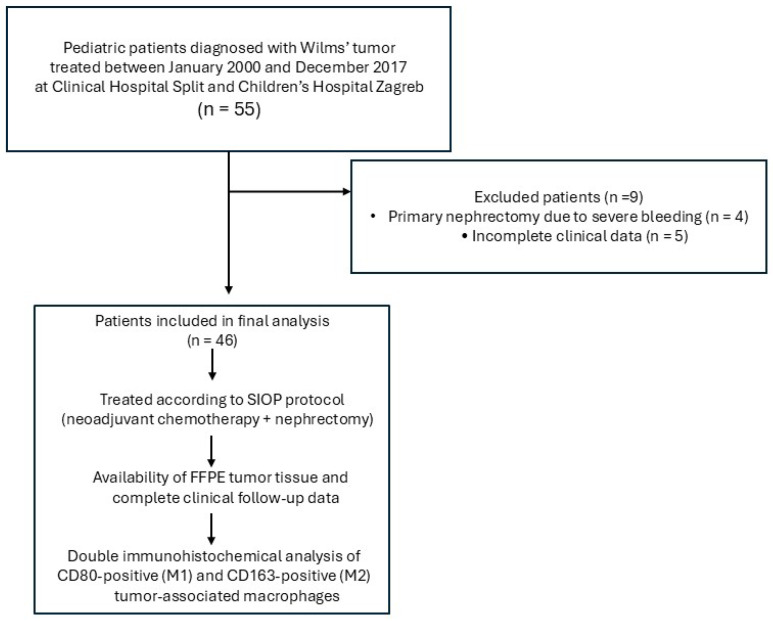
Flow diagram illustrating patient selection and study design. A total of 55 pediatric patients diagnosed with Wilms’ tumor were initially identified. Nine patients were excluded due to primary nephrectomy caused by severe bleeding (*n* = 4) or incomplete clinical data (*n* = 5). The final study cohort consisted of 46 patients treated according to the SIOP-RTSG protocol, which includes preoperative (neoadjuvant) chemotherapy followed by nephrectomy. Tumor specimens were obtained at the time of surgery for histopathological and immunohistochemical analysis. Postoperative chemotherapy, with or without radiotherapy depending on tumor stage and histological risk group, was subsequently administered according to SIOP guidelines. Tumor-associated macrophage subpopulations were analyzed using double immunohistochemistry.

A flow diagram illustrating the study design is presented in Figure 1.

### 2.2. Clinical and Pathohistological Data

Clinical data collected from medical records included patient age and sex, presenting clinical signs, congenital anomalies, and laboratory parameters, including urea, creatinine, lactate dehydrogenase (LDH), ferritin, neuron-specific enolase (NSE), alpha-fetoprotein (AFP), beta-human chorionic gonadotropin (β-hCG), and urinalysis results. Only hematuria and/or proteinuria were considered as significant pathological urinalysis results. Additional clinical variables comprised tumor stage, tumor site and volume, number of resected lymph nodes, type of neoadjuvant chemotherapy and treatment protocol (SIOP 2001 or SIOP 2014), administration of radiotherapy, tumor progression, presence of metastases, and survival outcomes.

Pathohistological data were obtained from formal histopathological reports and included histological tumor subtype (favorable or unfavorable), risk group classification, largest tumor diameter, number of metastatic lymph nodes, presence of renal capsule invasion, tumor focality, resection margin status, renal vein thrombosis, and the percentage of tumor necrosis.

Tumor histology was further subclassified into mixed, blastemal, epithelial, regressive, anaplastic (diffuse anaplasia), completely necrotic, and tumors composed predominantly of a single component. The presence of nephrogenic rests was also recorded. Tumor volume prior to neoadjuvant chemotherapy was calculated using pre-treatment computed tomography measurements according to the formula: length × depth × thickness × 0.523.

Patients were stratified by age into two groups (<24 months and ≥24 months) and were followed from the time of diagnosis throughout treatment and for a five-year follow-up period.

### 2.3. Immunohistochemistry

Double immunohistochemical staining was performed using the UltraView Universal Alkaline Phosphatase Red Detection Kit and the UltraView Universal DAB Detection Kit (Ventana Medical Systems, Tucson, AZ, USA). Representative tumor tissue sections were processed on an automated staining platform (Ventana BenchMark ULTRA IHC/ISH Staining Module; Ventana Roche, Tucson, AZ, USA) according to the manufacturer’s instructions.

**Figure 2:**
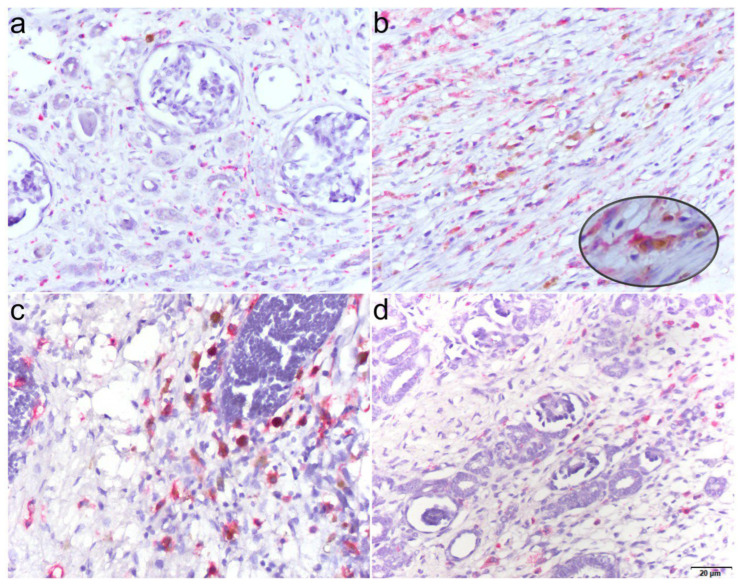
Histological images depicting the distribution of M1 and M2 macrophages in normal renal tissue and Wilms’ tumor samples. Double immunohistochemical staining was used to mark CD80 positive (brown) M1 macrophages and CD163 positive (red) M2 macrophages. Low M1 and M2 macrophage density was present in normal renal tissue (**a**), while a high density was visible in areas of tumor regression. Insertion: CD80 positive (brown) M1 macrophages and CD163 positive (red) M2 macrophages (magnification of 400×) (**b**). M1 and M2 macrophages were present in the tumor stroma as well as in blastemal (**c**) and epithelial components (**d**). M2 macrophages were in general more abundant within the tumor microenvironment compared to M1 macrophages. All images were taken at a total magnification of 200×.

To distinguish macrophage subpopulations, a rabbit anti-CD80 antibody (Abcam, Cambridge, Cambridgeshire, United Kingdom; dilution 1:1000) and a ready-to-use mouse anti-CD163 antibody (Ventana Medical Systems, Tuscon, AZ, USA) were used to identify M1 and M2 macrophages, respectively. Brown cytoplasmic staining (DAB) was considered indicative of CD80-positive M1 macrophages, whereas red cytoplasmic staining (alkaline phosphatase) was considered positive for CD163-expressing M2 macrophages (Figure 2).

### 2.4. TAM Quantification

TAMs in WT specimens were quantified by enumerating CD80-positive (M1) and CD163-positive (M2) macrophages. For each sample, positively stained cells were quantified within a standardized area of 1 mm^2^, corresponding to four high-power fields (HPFs; 400× total magnification) using an Olympus BX46 microscope (Olympus Corporation, Hachioji, Tokyo, Japan) equipped with a 40× objective (field number 22). Areas with the highest macrophage density were selected for analysis due to a substantial proportion of the tumors containing extensive necrotic regions following neoadjuvant chemotherapy, making random field selection unsuitable for reliable immune cell quantification.

The absolute numbers of M1 and M2 TAMs, as well as the M1/M2 ratio, were calculated and included in subsequent statistical analyses. To evaluate the association between TAM density and clinical or pathohistological parameters, tumor samples were classified as positive or negative for M1 and M2 TAM infiltration using a threshold-based approach as previously described by Tian et al. [36]. Briefly, the median values of M1 and M2 TAM counts across all samples were calculated and used as cutoff thresholds. Samples with TAM counts below the median were classified as negative, whereas those with counts equal to or above the median were classified as positive for the respective macrophage subtype. The same classification strategy was applied independently for M1 and M2 TAMs. The median cutoff threshold was selected specifically to allow for direct comparison with the results of Tian et al. [36], which have performed a similar analysis on WT samples treated according to the COG protocol.

### 2.5. Statistical Analysis

All statistical analyses were performed using GraphPad Prism software (version 9.0.0; GraphPad Software, San Diego, CA, USA). Continuous variables are presented as means ± standard deviation (SD). Data distribution was assessed using the Shapiro–Wilk test. The Mann–Whitney U test was applied to evaluate differences in total TAM counts and M1/M2 ratios between patient groups stratified according to clinical and pathohistological characteristics. Associations between categorical variables were analyzed using Pearson’s chi-squared test. *P*-values less than 0.05 were considered statistically significant.

## 3. Results

### 3.1. Patient Characteristics and Tumor Features

A total of 46 pediatric patients were included in the final analysis after application of the inclusion and exclusion criteria. Clinical characteristics and laboratory findings are summarized in Table 1. The median age at diagnosis was 36.5 months (interquartile range, 15.75–54.75 months); nineteen patients (41.3%) were male, metastatic disease was present in 11 patients (23.9%), eight patients (17.4%) had bilateral tumors, and congenital anomalies were identified in 8 patients (17.4%).

All patients received neoadjuvant chemotherapy according to the SIOP–RTSG protocol. The SIOP 2001 protocol was applied in 37 patients (80.4%), whereas 9 patients (19.6%) were treated according to the SIOP 2014 protocol.

The mean tumor volume prior to neoadjuvant chemotherapy was 333.66 ± 286.89 mL. Tumors smaller than 500 mL accounted for 76.1% of cases, while 23.9% had a volume ≥ 500 mL. Most tumors were unifocal (89.1%) and classified as stage I following pathohistological evaluation (65.2%). Necrosis involving less than 60% of the tumor mass was observed in 35 tumors (76.1%), whereas three tumors (6.5%) were classified as low-risk WT with more than 90% tumor necrosis.

The most frequent histological subtype was mixed (45.7%), followed by regressive (17.4%), blastemal (15.2%), epithelial (10.9%), and stromal (8.7%) subtypes. Only one tumor exhibited diffuse anaplasia. Overall, 34 patients (73.9%) were classified as intermediate risk and 9 patients (19.6%) as high risk. One tumor initially classified as low risk was bilateral; however, the contralateral tumor demonstrated a blastemal pattern and was therefore reclassified as high risk.

### 3.2. Survival and Prognostic Factors

The five-year overall survival and event-free survival rates were both 94.5%, comparable to published WHO data, with a slightly lower mortality rate. Three patients died of WT: two with stage III disease and one with stage V disease. Two of these patients had tumor volumes ≥ 500 mL, while one had a tumor volume < 500 mL. Histologically, two tumors were blastemal, and one was anaplastic. Two patients died following relapse with pulmonary metastases, while one patient died from widely metastatic disease involving the lungs, liver, and bones.

Due to the limited number of events, survival analysis was not feasible. Instead, established prognostic factors—including age at diagnosis, tumor stage, tumor volume, risk group, and histological subtype—were used as surrogate indicators of prognosis. No significant associations were observed between patient age or tumor volume and serum tumor marker levels. Advanced tumor stage was significantly associated with elevated LDH levels (χ^2^ = 8.095, *p* = 0.004) and increased creatinine levels (χ^2^ = 5.014, *p* = 0.025). Unfavorable histology was associated with decreased serum urea levels (χ^2^ = 3.937, *p* = 0.047).

### 3.3. TAM Infiltration

**Figure 3:**
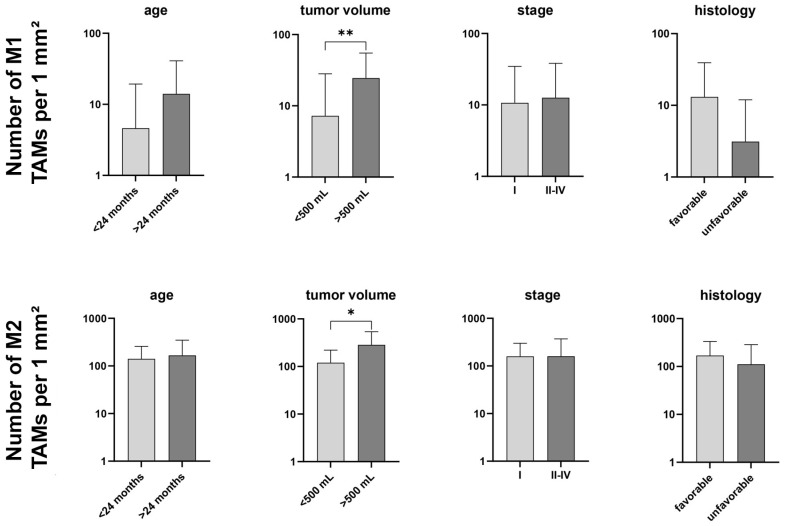
Graphs depicting the total number of M1 and M2 tumor-associated macrophages in Wilms tumor samples segregated by known prognostic factors (patient’s age at time of diagnosis, tumor volume, tumor stage, and tumor histology). Data are presented as the mean ± standard deviation (vertical line) and were analyzed using the Mann–Whitney U test. Significant differences are indicated by * *p* < 0.05 and ** *p* < 0.01.

Analysis of total TAMs revealed a significantly higher number of TAMs in tumors with a volume ≥ 500 mL (*p* = 0.033) and in unifocal tumors (*p* = 0.012) (Table 2). No statistically significant differences in total TAM counts were observed for other analyzed clinicopathological variables (Table 2, Table 3 and Table 4) (Figure 3).

When M1 and M2 macrophages were analyzed separately, tumors with a volume ≥ 500 mL demonstrated significantly higher numbers of M1 macrophages (*p* = 0.006) and a trend for higher numbers of M2 macrophages (*p* = 0.084) (Table 5). No significant differences were detected with respect to age at diagnosis, tumor stage, or histological subtype (Table 5, Table 6 and Table 7).

Overall, WTs treated with neoadjuvant chemotherapy exhibited significantly lower infiltration by M1 macrophages compared with M2 macrophages. M2 macrophages were the predominant macrophage population within the TME. In contrast, TAMs were absent in nephrogenic rests and were only rarely observed in adjacent normal renal parenchyma.

### 3.4. M1/M2 Ratio and Associations with Clinicopathological Parameters

The M1/M2 ratio was significantly higher in tumors with a volume ≥ 500 mL (*p* = 0.020) (Table 2) and in patients with elevated serum creatinine (*p* = 0.008) and NSE levels (*p* = 0.027) compared with patients with normal creatinine and NSE values, respectively (Table 4). No significant differences in the M1/M2 ratio were observed for other clinicopathological characteristics.

Patients with regressive tumors exhibited a significantly higher M1/M2 ratio (0.152 ± 0.140) compared with those with mixed (0.038 ± 0.102; *p* = 0.007), epithelial (0; *p* = 0.003), and blastemal tumors (0.007 ± 0.018; *p* = 0.006). No significant association was found between total TAM counts and specific histological subtypes. However, the M1/M2 ratio showed a strong positive correlation with total TAM numbers (r = 0.580, *p* < 0.001).

Tumor M1 positivity was significantly associated with tumor volume ≥ 500 mL (χ^2^ = 7.527, *p* = 0.006), elevated serum NSE levels (χ^2^ = 4.212, *p* = 0.040), and increased serum creatinine levels (χ^2^ = 6.938, *p* = 0.0008) (Table 5 and Table 7). Tumor M2 positivity was significantly associated with unifocal tumor presentation (χ^2^ = 5.610, *p* = 0.018) (Table 5). No additional significant associations were observed.

## 4. Discussion

WT represents one of the most successful examples of multimodal cancer treatment in pediatric oncology, with overall survival rates approaching 90% [37,38]. The combination of surgery, chemotherapy, and, in selected high-risk cases, radiotherapy has led to excellent outcomes, particularly in patients with favorable-histology WT, while survival remains lower in patients with bilateral disease or high-risk histological subtypes [24]. Nevertheless, approximately 10–15% of children develop high-risk WT characterized by unfavorable histology, including diffuse anaplasia or blastemal predominance, which is associated with lower survival rates, treatment resistance, and relapse [39,40,41]. These challenges have driven ongoing efforts to identify novel biomarkers that could refine risk stratification, reduce treatment-related toxicity, and improve outcomes in high-risk patients.

According to the current SIOP-RTSG risk stratification system, established prognostic factors include patient age and tumor volume after preoperative chemotherapy, tumor stage before and after preoperative chemotherapy, post-chemotherapy histology, and lung nodule response in stage IV disease [39]. In our cohort, the median age at diagnosis was 36.5 months, consistent with previous reports indicating that WT most commonly presents after the first year of life [42,43,44]. Large-scale analyses of SIOP-93-01 and SIOP-2001 cohorts have demonstrated that older age at diagnosis correlates with higher tumor stage, increased frequency of high-risk histological subtypes, larger tumor volume, and higher relapse rates [42,43], supporting age as a robust prognostic parameter.

Tumor volume before neoadjuvant chemotherapy is another important prognostic indicator within the SIOP framework, with a cutoff value of ≥500 mL identifying patients at increased risk [45,46]. In the present study, most tumors were smaller than 500 mL and were classified as intermediate risk, reflecting a cohort with generally favorable baseline characteristics. Histologically, tumors exhibiting diffuse anaplasia or persistent blastemal predominance after neoadjuvant chemotherapy are considered high risk and are more frequently observed in advanced-stage disease [43,47,48]. Persistence of blastemal components following chemotherapy is associated with treatment resistance and poor outcome [39,41,45,46,49]. In our cohort, blastemal histology was predominantly observed in children older than 24 months, and deaths occurred exclusively among patients with blastemal or anaplastic subtypes, underscoring the combined prognostic impact of age and histology.

Beyond tumor-intrinsic features, increasing attention has been directed toward the TME as a critical determinant of tumor progression and therapeutic response. The TME consists of immune cells, vasculature, extracellular matrix, and stromal components that collectively support tumor growth, invasion, and metastasis. During early tumorigenesis, classically activated M1 macrophages may act as first-line immune responders, exerting antitumor activity [50,51]. However, dynamic changes within the TME—such as hypoxia, altered glucose availability, and pH shifts—promote macrophage polarization toward the alternatively activated, immunosuppressive M2 phenotype [49,50,51,52,53]. M2 macrophages facilitate tissue remodeling, angiogenesis, and immune evasion, ultimately impairing antitumor immunity and reducing responsiveness to chemotherapy, radiotherapy, and immunotherapy [52,54].

The prognostic relevance of TAMs in WT remains incompletely defined, particularly in the context of neoadjuvant chemotherapy. Most previous studies have analyzed TAMs in primarily resected WT specimens treated according to the COG protocol [36,53,54,55]. Tian et al. demonstrated increased M1 and M2 macrophage density in tumor tissue compared with adjacent normal kidney tissue, with M2 macrophages enriched in higher-stage tumors [36]. Similarly, Liou et al. reported correlations between macrophage infiltration, tumor stage, and microvascular invasion in largely untreated WT samples [53].

In contrast, our study focused exclusively on WT specimens treated with neoadjuvant chemotherapy according to the SIOP protocol. We observed that both M1 and M2 macrophages were generally scarce in viable tumor tissue following chemotherapy, with markedly lower infiltration by M1 macrophages and predominance of M2 macrophages within the TME. Importantly, TAMs were largely absent from nephrogenic rests and only rarely detected in adjacent normal renal parenchyma, suggesting a tumor-specific microenvironmental response. The relatively low TAM density observed in our cohort compared with reports in untreated WT may reflect the impact of neoadjuvant chemotherapy on the TME. Chemotherapy may reduce overall macrophage infiltration and/or modulate macrophage polarization, although the absence of paired pre- and post-treatment samples precludes definitive conclusions. Notably, TAMs were absent in nephrogenic rests, suggesting that macrophage recruitment is associated with malignant transformation rather than the pre-neoplastic state and may be driven by tumor-specific signaling mechanisms. The association between a higher M1/M2 ratio and regressive histology further supports the hypothesis that macrophage polarization may reflect chemotherapy responsiveness. If validated in larger studies, TAM profiling could potentially complement existing risk stratification models; however, these findings remain exploratory and require prospective confirmation.

Quantitative analysis revealed a significantly higher total TAM count in tumors with a volume ≥ 500 mL and in unifocal tumors, whereas no significant associations were found between TAM density and tumor stage or histological subtype. It should be emphasized that findings concerning histological subgroups and tumor focality should be interpreted cautiously, given the limited sample size of the study cohort. When macrophage subpopulations were analyzed separately, both M1 and M2 macrophages were more abundant in larger tumors, supporting the notion that tumor burden influences immune cell recruitment. The M1/M2 ratio correlated positively with total TAM numbers and was significantly higher in tumors with larger volume, as well as in patients with elevated serum NSE and creatinine levels. Although no WT-specific serum biomarkers currently exist, these associations may reflect more aggressive tumor biology and altered metabolic or inflammatory states.

Notably, the regressive histological subtype exhibited a significantly higher M1/M2 ratio compared with mixed, epithelial, and blastemal subtypes. This observation is particularly relevant, as regressive tumors reflect a favorable response to neoadjuvant chemotherapy. The predominance of M1 macrophages within these tumors may indicate that a more pro-inflammatory microenvironment contributes to enhanced chemosensitivity and tumor regression. In this context, an increased M1/M2 ratio could represent not only a marker of immune activation but also a potential indicator of therapeutic responsiveness. Although the stromal and connective tissue components characteristic of regressive tumors may partly facilitate M1 infiltration, the association between M1 predominance and tumor regression suggests a biologically meaningful interaction between macrophage polarization and treatment response. Further studies are warranted to determine whether modulation of TAM polarization could enhance chemotherapy efficacy in WT.

In contrast to previous reports, we did not observe significant differences in macrophage distribution across tumor stages. This discrepancy may reflect the limited sample size, but it also suggests that neoadjuvant chemotherapy itself may modulate TAM composition, potentially masking stage-dependent differences observed in untreated tumors [36].

Our findings are in line with emerging evidence highlighting complex interactions between immune cells and WT components. Maturu et al. demonstrated that TAMs infiltrate both stromal and epithelial tumor compartments and that inflammatory marker expression correlates with macrophage density [54]. Sherif et al. showed that macrophage infiltration in untreated WT is associated with angiogenic signaling and unfavorable prognosis [55]. Notably, Fiore et al. provided evidence that WT blastemal and epithelial components can suppress natural killer cell activity and promote M2 polarization, contributing to an immunosuppressive TME and immune escape, even following neoadjuvant chemotherapy [38]. In this context, a recent study by Wang et al. reported findings that are largely consistent with our observations. The authors investigated TAM infiltration in WT specimens from patients treated with either preoperative neoadjuvant chemotherapy or chemotherapy administered after surgery. They demonstrated a high recruitment of TAMs in WT tissues, a positive correlation between TAM and M2 macrophage infiltration and tumor stage, and an association between increased M2 macrophage presence following chemotherapy and poorer prognosis. Although their study differed from ours in terms of treatment heterogeneity, methodology, and inclusion of survival analysis, both studies support the concept that TAM polarization—particularly M2 predominance—plays a significant role in WT progression and may represent a clinically relevant target for prognostic stratification and therapeutic modulation [56]. Together, these findings support the concept that the WT TME may serve as a biologically relevant prognostic and therapeutic target. Similar patterns of macrophage polarization have been described across a range of pediatric solid tumors. In pediatric brain tumors, TAMs frequently display an M2-like phenotype, contributing to the development of an immunosuppressive, “immune-cold” microenvironment that limits effective anti-tumor immune responses and reduces responsiveness to immunotherapy. In medulloblastoma, M2 polarization is particularly prominent in SHH tumors, whereas pediatric high-grade gliomas are uniformly characterized by a macrophage-dominated, M2-skewed microenvironment that suppresses T-cell activity and promotes tumor progression and therapy resistance. Likewise, in retinoblastoma, osteosarcoma, hepatoblastoma, and high-risk neuroblastoma, M2-polarized macrophages contribute to immune evasion, angiogenesis, tumor growth, and reduced chemotherapy efficacy through tumor–immune signaling interactions and immunosuppressive cytokine production. Collectively, these findings reinforce the concept that macrophage polarization represents a central mechanism of immune modulation in pediatric malignancies and further support its potential as a prognostic biomarker and therapeutic target in WT [57,58,59,60,61].

The present study has several limitations. The relatively small cohort size and inclusion of patients from only two centers may limit the generalizability of our findings. We were unable to perform multivariate analyses due to the limited sample size. Therefore, it cannot be excluded that the observed associations between our parameters are at least partially dependent. Prospective studies with larger cohorts should examine whether these associations remain independent after such adjustment. Additionally, TAM quantification relied on a single immunohistochemical approach. Future studies should apply complementary methodologies, such as multiplex immunohistochemistry with additional M1 (CD86, iNOS) and M2 (CD203, CD204) macrophage markers or transcriptomic profiling, to better characterize macrophage phenotypes. Direct comparisons between WT treated with and without neoadjuvant chemotherapy would be particularly informative in elucidating therapy-induced changes in the TME. Likewise, survival analysis was not performed due to the low number of events in our cohort; therefore, future studies including larger patient populations and longer follow-up periods are needed to adequately assess the prognostic relevance of TAM polarization in WT.

Despite these limitations, our study provides novel insight into TAM distribution in neoadjuvantly treated WT, a setting that reflects current clinical practice in most SIOP-affiliated countries. Given the persistent risk of relapse in high-risk WT and the emergence of pharmacological strategies targeting M2 macrophages [62,63,64], further exploration of TAMs as prognostic biomarkers and therapeutic targets is warranted.

## 5. Conclusions

In this study, we characterized the distribution of M1 and M2 macrophages in WT following neoadjuvant chemotherapy and demonstrated low overall macrophage infiltration with a predominance of M2-polarized cells. We further showed that TAM polarization is associated with tumor volume, selected biochemical parameters, and histological subtype. These findings suggest that macrophage composition may reflect tumor biology and treatment responsiveness in the post-neoadjuvant setting. Future studies should validate these observations in larger, independent cohorts with extended follow-up and incorporate functional analyses to clarify the mechanistic role of M2 macrophages in chemotherapy response and potential treatment resistance. A deeper understanding of TAM biology in this context may ultimately support the development of novel prognostic biomarkers and macrophage-targeted therapeutic strategies in WT.

## Figures and Tables

**Table 1:** Clinical characteristics and laboratory findings of the study cohort (*n* = 46).

Variable	Findings
Age (months)	<24: 13 (28.3%); ≥24: 33 (71.7%)
Sex	Male: 19 (41.3%); Female: 27 (58.7%)
Clinical presentation *	Palpable mass: 15 (32.6%); Hematuria: 2 (4.3%); Abdominal pain: 1 (2.2%)
Tumor laterality	Right: 17 (37.0%); Left: 21 (45.7%); Bilateral: 8 (17.4%)
Urea (mmol/L)	Normal: 31 (67.4%); Decreased: 15 (32.6%)
Creatinine (µmol/L) **	Normal: 37 (80.4%); Increased: 9 (19.6%)
LDH (U/L)	Normal: 12 (26.1%); Increased: 26 (56.5%); Not sampled: 8 (17.4%)
Ferritin (ng/mL)	Normal: 20 (43.5%); Increased: 14 (30.4%); Not sampled: 12 (26.1%)
Urinalysis	Normal: 35 (76.1%); Pathological: 11 (23.9%)
NSE (ng/mL)	Normal: 20 (43.5%); Increased: 15 (32.6%); Not sampled: 11 (23.9%)
AFP (ng/mL)	Normal: 20 (43.5%); Increased: 6 (13.0%); Not sampled: 20 (43.5%)

* Patients may have presented with more than one clinical symptom; therefore, percentages for clinical presentation do not sum to 100%. ** All patients with increased values of creatinine had a decreased estimated glomerular filtration rate (eGFR) according to the Bedside Schwartz equation. Abbreviations: LDH, lactate dehydrogenase; NSE, neuron-specific enolase; AFP, alpha-fetoprotein.

**Table 2:** Association of pathohistological factors with total tumor-associated macrophage (TAM) counts and M1/M2 ratio.

Pathohistological Factor	Category (*n*)	Total TAMs (Mean ± SD)	*p*	M1/M2 Ratio (Mean ± SD)	*p*
Tumor volume	<500 mL (35)	127.0 ± 107.8	0.033 *	0.048 ± 0.130	0.020 *
	≥500 mL (11)	308.7 ± 275.9		0.078 ± 0.085	
Tumor focality	Unifocal (41)	185.8 ± 183.0	0.012 *	0.062 ± 0.126	0.230
	Multifocal (5)	44.8 ± 33.5		0	
Tumor necrosis	<60% (35)	171.9 ± 198.3	0.376	0.042 ± 0.112	0.152
	≥60% (11)	166.0 ± 97.2		0.099 ± 0.141	
Tumor histology	Favorable (38)	182.3 ± 177.6	0.086	0.066 ± 0.130	0.201
	Unfavorable (8)	114.1 ± 182.9		0.006 ± 0.016	
Risk group	Intermediate (34)	175.2 ± 184.3	0.271	0.050 ± 0.117	0.669
	High (9)	133.3 ± 180.5		0.021 ± 0.048	

Data are presented as mean ± standard deviation (SD). *p*-values were calculated using the Mann–Whitney U test. * Statistically significant *p*-values.

**Table 3:** Association of demographic and clinical parameters with total tumor-associated macrophage (TAM) counts and M1/M2 ratio.

Clinical Parameter	Category (*n*)	Total TAMs (Mean ± SD)	*p*	M1/M2 Ratio (Mean ± SD)	*p*
Age	<24 months (13)	145.3 ± 121.6	0.750	0.020 ± 0.062	0.170
	≥24 months (33)	180.4 ± 197.1		0.069 ± 0.135	
Sex	Male (19)	202.9 ± 227.7	0.577	0.067 ± 0.144	0.878
	Female (27)	147.6 ± 133.7		0.047 ± 0.103	
Tumor stage	I (30)	169.5 ± 147.0	0.289	0.054 ± 0.125	0.552
	II–IV (16)	172.2 ± 231.8		0.058 ± 0.116	
Metastases	Yes (11)	233.8 ± 258.6	0.426	0.045 ± 0.068	0.391
	No (35)	150.5 ± 144.0		0.059 ± 0.133	
Congenital anomalies	Yes (8)	137.0 ± 111.8	0.782	0.025 ± 0.052	0.685
	No (38)	177.5 ± 189.9		0.062 ± 0.130	
Chemotherapy protocol	SIOP 2001 (37)	156.6 ± 153.7	0.474	0.055 ± 0.128	0.801
	SIOP 2014 (9)	227.2 ± 261.2		0.055 ± 0.086	

Data are presented as mean ± standard deviation (SD). *p*-values were calculated using the Mann–Whitney U test.

**Table 4:** Association of laboratory parameters with total tumor-associated macrophage (TAM) counts and M1/M2 ratio.

Laboratory Parameter	Category (*n*)	Total TAMs (Mean ± SD)	*p*	M1/M2 Ratio (Mean ± SD)	*p*
LDH	Normal (12)	152.9 ± 179.1	0.659	0.036 ± 0.118	0.254
	Increased (26)	183.7 ± 196.8		0.077 ± 0.137	
Ferritin	Normal (20)	156.8 ± 132.5	0.736	0.067 ± 0.145	0.653
	Increased (14)	171.8 ± 188.2		0.069 ± 0.130	
NSE	Normal (20)	143.2 ± 175.3	0.156	0.024 ± 0.092	0.027 *
	Increased (15)	220.0 ± 215.3		0.124 ± 0.164	
AFP	Normal (20)	178.9 ± 187.1	0.941	0.063 ± 0.140	0.708
	Increased (6)	123.3 ± 96.8		0.114 ± 0.186	
Urea	Normal (31)	147.0 ± 137.2	0.590	0.065 ± 0.139	0.690
	Decreased (15)	219.0 ± 241.3		0.036 ± 0.070	
Creatinine	Normal (37)	173.1 ± 192.2	0.698	0.035 ± 0.091	0.008 *
	Increased (9)	159.6 ± 112.4		0.138 ± 0.187	
Urinalysis	Normal (35)	173.5 ± 196.9	0.404	0.044 ± 0.107	0.468
	Pathological (11)	160.7 ± 105.9		0.091 ± 0.155	

Data are presented as mean ± standard deviation (SD). *p*-values were calculated using the Mann–Whitney U test. * Statistically significant *p*-values.

**Table 5:** Association of pathohistological factors with M1 and M2 tumor-associated macrophage (TAM) positivity.

Factor	Category (*n*)	M1 TAMs Positive, *n* (%)	*p*	M2 TAMs Positive, *n* (%)	*p*
Tumor volume	<500 mL (35)	7 (20.0)	0.006 *	15 (42.9)	0.084
	≥500 mL (11)	7 (63.6)		8 (72.7)	
Tumor focality	Unifocal (41)	14 (34.1)	0.117	23 (56.1)	0.018 *
	Multifocal (5)	0 (0)		0 (0)	
Tumor necrosis	<60% (35)	9 (25.7)	0.215	16 (45.7)	0.299
	≥60% (11)	5 (45.5)		7 (63.6)	
Tumor histology	Favorable (38)	13 (34.2)	0.225	21 (55.3)	0.120
	Unfavorable (8)	1 (12.5)		2 (25.0)	
Risk group	Intermediate (34)	10 (29.4)	0.669	18 (52.9)	0.295
	High (9)	2 (22.2)		3 (33.3)	

Macrophage positivity was defined using median cutoff values for M1 and M2 tumor-associated macrophages. Associations were analyzed using Pearson’s chi-squared test. * Statistically significant *p*-values.

**Table 6:** Association of demographic and disease-related clinical parameters with M1 and M2 tumor-associated macrophage (TAM) positivity.

Clinical Parameter	Category (*n*)	M1 TAMs Positive, *n* (%)	*p*	M2 TAMs Positive, *n* (%)	*p*
Age	<24 months (13)	2 (15.4)	0.164	6 (46.2)	0.743
	≥24 months (33)	12 (36.4)		17 (51.5)	
Sex	Male (19)	6 (31.6)	0.888	11 (57.9)	0.369
	Female (27)	8 (29.6)		12 (44.4)	
Tumor stage	I (30)	8 (26.7)	0.447	18 (60.0)	0.063
	II–IV (16)	6 (37.5)		5 (31.3)	
Metastases	Yes (11)	5 (45.5)	0.215	5 (45.5)	0.730
	No (35)	9 (25.7)		18 (51.4)	
Congenital anomalies	Yes (8)	2 (25.0)	0.713	3 (37.5)	0.437
	No (38)	12 (31.6)		20 (52.6)	
Chemotherapy protocol	SIOP 2001 (37)	11 (29.7)	0.833	18 (48.6)	0.710
	SIOP 2014 (9)	3 (33.3)		5 (55.6)	

Macrophage positivity was defined using median cutoff values for M1 and M2 tumor-associated macrophages. Associations were analyzed using Pearson’s chi-squared test.

**Table 7:** Association of laboratory parameters with M1 and M2 tumor-associated macrophage (TAM) positivity.

Laboratory Parameter	Category (*n*)	M1 TAMs Positive, *n* (%)	*p*	M2 TAMs Positive, *n* (%)	*p*
LDH	Normal (12)	2 (16.7)	0.257	6 (50.0)	0.825
	Increased (26)	9 (34.6)		12 (46.2)	
Ferritin	Normal (20)	5 (25.0)	0.499	11 (55.0)	0.268
	Increased (14)	5 (35.7)		5 (35.7)	
NSE	Normal (20)	3 (15.0)	0.040 *	9 (45.0)	0.625
	Increased (15)	7 (46.7)		8 (53.3)	
AFP	Normal (20)	6 (30.0)	0.877	8 (40.0)	0.250
	Increased (6)	2 (33.3)		4 (66.7)	
Urea	Normal (31)	10 (32.3)	0.699	16 (51.6)	0.753
	Decreased (15)	4 (26.7)		7 (46.7)	
Creatinine	Normal (37)	8 (21.6)	0.008 *	18 (48.6)	0.710
	Increased (9)	6 (66.7)		5 (55.6)	

Macrophage positivity was defined using median cutoff values for M1 and M2 tumor-associated macrophages. Associations were analyzed using Pearson’s chi-squared test. * Statistically significant *p*-values.

## Data Availability

The original contributions presented in this study are included in the article. Further inquiries can be directed to the corresponding author.

## Abbreviations

The following abbreviations are used in this manuscript:

WT	Wilms’ tumor
TAM	Tumor-associated macrophages
SIOP	International Society of Pediatric Oncology
COG	Children’s Oncology Group
NWTSG	National Wilms Tumor Study Group
TME	Tumor microenvironment
FFPE	Formalin-fixed, paraffin-embedded
LDH	Lactate dehydrogenase
NSE	Neuron-specific enolase
AFP	Alpha-fetoprotein
β-hCG	beta-human chorionic gonadotropin
